# DKK3–CKAP4 signaling drives fibroimmune remodeling and hair follicle miniaturization in androgenetic alopecia

**DOI:** 10.7150/thno.135412

**Published:** 2026-05-29

**Authors:** Seungchan An, Hyunju Kim, Mei Zheng, In Guk Park, Leegu Song, Jino Kim, Minsoo Noh, Jong-Hyuk Sung

**Affiliations:** 1 Epi Biotech Co., Ltd., Incheon 21984, Republic of Korea.; 2 Natural Products Research Institute, College of Pharmacy, Seoul National University, Seoul 08826, Republic of Korea.; 3 Ludwig Institute for Cancer Research, Princeton University, Princeton, NJ 08544, USA.; 4 New Hair Plastic Surgery Clinic, Seoul, Republic of Korea.

**Keywords:** androgenetic alopecia, dermal papilla cells, fibroimmune remodeling, DKK3–CKAP4 signaling, epithelial–mesenchymal crosstalk.

## Abstract

**Rationale:**

Androgenetic alopecia (AGA) is characterized by progressive hair follicle miniaturization driven by androgen signaling and alterations in the follicular microenvironment, including fibrosis and inflammation. Therefore, identifying key mediators of epithelial–mesenchymal crosstalk is essential for elucidating disease mechanisms and developing targeted therapeutic strategies. In this context, we investigated the role of the DKK3–CKAP4 signaling axis as a potential mediator of fibroimmune remodeling in AGA and evaluated its functional and therapeutic relevance.

**Methods:**

We performed meta-analysis of human and mouse single-cell RNA sequencing datasets to identify candidate signaling pathways associated with AGA. Spatial expression of DKK3 and its receptor CKAP4 was validated by immunostaining. Functional roles were assessed using hair follicle organ culture and androgen-induced AGA mouse models, with pathway modulation by recombinant DKK3 and neutralizing antibodies. Single-cell transcriptomic analyses were conducted to characterize cellular and molecular changes following pathway inhibition.

**Results:**

DKK3 was expressed in epithelial and mesenchymal compartments of the hair follicle, whereas CKAP4 was enriched in dermal papilla cells and dermal fibroblasts. Recombinant DKK3 suppressed hair follicle growth in organ culture, while neutralization of DKK3 or CKAP4 restored hair elongation and promoted hair regeneration in androgen-driven models. Single-cell analysis revealed that androgen signaling induced fibroimmune transcriptional program in mesenchymal cells, characterized by extracellular matrix deposition and inflammatory pathway activation. Inhibition of DKK3 attenuated these responses and partially restored dermal papilla cell populations.

**Conclusions:**

These findings identify the DKK3–CKAP4 axis that contributes to fibroimmune remodeling in AGA. Targeting this pathway restores a regenerative follicular microenvironment and represents a potential therapeutic strategy for reversing hair follicle miniaturization.

## Introduction

Androgenetic alopecia (AGA), the most prevalent form of hair loss in adults, is characterized by progressive miniaturization of hair follicles (HFs) in a patterned distribution. This condition is driven primarily by excessive androgen signaling‒particularly dihydrotestosterone (DHT) acting via the androgen receptor (AR)‒which affects dermal papilla cells (DPCs) and their ability to sustain the anagen phase of hair growth 1-3. However, beyond androgen signaling, increasing evidence suggests that AGA involves aberrant modulation of key morphogenetic pathways and local inflammatory and fibrotic remodeling of the scalp microenvironment 4, 5.

Among the most well-characterized molecular mediators in AGA is Dickkopf-1 (DKK1), a secreted inhibitor of canonical Wnt/β-catenin signaling 6-8. DKK1 expression is reportedly upregulated in DPCs following DHT stimulation and has been shown to induce catagen-like changes in hair follicles both *in vitro* and *in vivo* 9-11. DKK1 signaling network appears to be involved in the pathogenesis of AGA, promoting a fibrotic, anti-proliferative, and immune activated milieu that sustains follicular miniaturization 12, 13. By antagonizing Wnt ligands through low-density lipoprotein receptor-related protein 5/6 (LRP5/6) receptor binding, DKK1 suppresses follicular stem cell activation, accelerates regression of the hair cycle, and impairs hair shaft elongation. Elevated levels of DKK1 have been reported in balding scalp regions contribute directly to follicular miniaturization and impaired regenerative capacity, positioning DKK1 as a central effector of androgen-driven hair loss 14, 15. However, we found in the previous study that DKK1 is not highly expressed in DPCs and outer-root sheath cells, but in adipose-derived stem cells surrounding hair follicle 8. On the contrary, Dickkopf-2 (DKK2) and DKK3 is highly expressed in mesenchymal cells such as DPCs and dermal fibroblasts (DFs) in hair microenvironment (Figure S1, GSE295410).

Of note, DKK3 protein becomes localized to the Wnt-inactive inner bulge during the hair cycle growth phase, and play a key role in reinforcing hair follicle stem cell differentiation 16. Genetic ablation of DKK3 significantly attenuates radiation-induced skin fibrosis and dermatitis, highlighting DKK3 as a potential therapeutic target for mitigating skin or hair damage 16, 17. In parallel, DKK3 has emerged as an additional contributor to hair follicle dysfunction 17. Unlike DKK1, DKK3 does not bind LRP5/6, but instead signals through cytoskeleton-associated protein 4 (CKAP4), a multifunctional receptor capable of mediating noncanonical Wnt, Akt, and NF-κB activation 18-20. DKK3 expression is induced under stress and injury conditions and has been implicated in tissue fibrosis, chronic inflammation, and immune evasion 21, 22. Notably, proteomic analyses have revealed that CKAP4 is significantly upregulated in DPCs from balding scalp, suggesting that the DKK3–CKAP4 axis may further propagate hair follicle atrophy by altering mesenchymal signaling responsiveness 23.

Given these findings, we hypothesize that the DKK3–CKAP4 signaling axis functions as a modulatory pathway in AGA and therapeutic inhibition of the DKK3/CKAP4 signaling axis-such as through monoclonal antibodies or receptor blockade may restore a pro-regenerative microenvironment conducive to hair follicle growth. In this study, we explore the pathogenic role of DKK3/CKAP4 signaling in AGA and propose a novel therapeutic framework for targeting this pathway to reverse or prevent follicular miniaturization.

## Materials and Methods

### Animals

Male adult (4 and 7 weeks old) C3H/HeN mice were purchased from Orient Bio Co. Ltd (Sungnam, Korea). All animal studies were conducted with the approval of the Animal Care and Use Committee of Yonsei University (IACUC-A-202403-1820-02).

### Telogen-to-anagen transition

Animal experiments were performed according to Kim 24 and Paus *et al*. 25. The dorsal area (2.5 cm × 4 cm) of 7-week-old, 22–24 g C3H/HeN mice, which were in the telogen stage of the hair cycle, were shaved using a hair clipper and hair removal cream (Ildong, Seoul, Korea). Subcutaneous injection of recombinant DKK3 protein (rDKK3; Sino Biological, Beijing, China) was administered for 10 days. Any darkening of the skin (indicative of hair cycle induction) was carefully monitored. After 13 days, dorsal hair was shaved and weighed.

### Hair organ culture

*Ex vivo* HF culture was performed using mouse vibrissae follicles and human HFs to evaluate hair shaft growth. Anagen-stage HFs were carefully isolated from the upper lip area of 4-week-old mice under a stereomicroscope. Individual HFs were maintained in serum-free Williams E medium containing L-glutamine (2 mM), insulin (10 μg/mL), hydrocortisone (10 ng/mL), penicillin (100 U/mL), and streptomycin (100 μg/mL). Images were captured at day 2 for mouse vibrissae and day 6 for human HFs, and hair shaft elongation was quantified from the images. Data are presented as the mean ± standard error (SE) from 10 HFs per group.

### Cell isolation and cell culture

Human HFs were obtained from New Hair Institute (South Korea) with written informed consent from all donors. All procedures involving human-derived samples were reviewed and approved by the relevant institutional ethical committee and conducted in accordance with the principles of the Declaration of Helsinki.

Human DPCs and dermal sheath cells (DSCs) were isolated from individual HFs under a stereomicroscope. Briefly, the follicle bulb was carefully inverted using fine forceps and needles to expose the dermal papilla region, and residual epithelial components were removed. The separated DP and DS were individually transferred onto CellBIND 35-mm culture dishes (Corning, USA) and allowed to attach under undisturbed conditions for approximately 10 days.

Human DPCs were cultured in follicle dermal papilla cell growth medium (PromoCell, Germany) with 1% antibiotic-antimycotic (Thermo Fisher Scientific, USA). Human DFs were kindly gifted by Prof. Sang Ho Oh (Severance Hospital, Yonsei University College of Medicine, Seoul, Korea) and cultured in Dulbecco’s modified Eagle’s medium (DMEM; Gibco, Germany) with 10% fetal bovine serum (FBS; Hyclone, USA), and maintained in a humidified incubator at 37 °C with 5% CO_2_.

### Cell proliferation assay

Human DPCs were plated in a 96-well plate at a density of 1500 cells/well and cultured for 24 h, and then followed by the indicated treatment. Cell viability was assessed by EZ-CYTOX cell viability assay kit (DoGEN, Seoul, Korea) according to manufacturer’s instruction. In short, EZ-CYTOX reaction mixture was added to culture medium and incubated for 1 hr. After brief mixing, absorbance of reaction product was measured by microplate reader (BMG Labtech, Germany) at 450nm wavelength.

### RNA extraction, cDNA synthesis, quantitative real-time PCR (qRT-PCR), and PCR array

Total RNA was extracted from cells using TRIzol Reagent (Thermo Fisher Scientific), followed by reverse transcription using a cDNA synthesis kit (Nanohelix, Daejeon, Korea). qRT-PCR was performed using the QuantSrudio1 Real-Time PCR System (Applied Biosystems, Foster City, CA, USA). The primer sequences used were as follows (forward and reverse, respectively): 5’-GATTCCCTGGACCTAAAGGTGC-3’ and 5’-AGCCTCTCCATCTTTGC-CAGCA-3’ for *COL1A1*; 5’- CCTGGTGCTAAAGGAGAAAGAGG-3’ and 5’-ATCACCAC-GACTTCCAGCAGGA-3’ for *COL1A2*; 5’- TGGTCTGCAAGGAATGCCTGGA-3’ and 5’-TCTTTCCCTGGGACACCATCAG-3’ for *COL3A1*; 5′-CATCACTGCCACCCAGAAGACT- G-3′ and 5′-ATGCCAGTGAGCTTCCCGTTCAG-3′ for *GAPDH*.

### Meta-analysis of scRNA-seq data

Publicly available scRNA-seq datasets from mouse (GSE295410) 4 and human (GSE212450) 26 studies were analyzed. Data processing was conducted using the Seurat R package (v5.0.3) following the previously described workflows 27, 28.

### AGA animal model

An androgen-induced alopecia model was established in 7-week-old male C3H mice (n = 30). Following dorsal hair removal, mice were subcutaneously administered testosterone propionate (TP; 0.5 mg/day) or DHT (1 mg/day) dissolved in corn oil (Sigma), five times per week. Anti-DKK3 antibody (25 μg/head; Bio X Cell, NH, USA) was administered subcutaneously once per week. Skin darkening was carefully monitored, and dorsal photographs were taken on day 19.

### Mouse skin scRNA-seq: tissue dissociation and library preparation

Dorsal skin samples were collected from control (Con), DHT-treated, and DHT plus anti-DKK3 antibody–treated mice, with four biologically independent mice per group (n = 4 mice per group). Hair was removed prior to tissue collection, and excised skin was trimmed to remove excess fat and connective tissue, minced, and processed into single-cell suspensions. Cells from four mice per group were pooled prior library preparation. Skin fragments were digested in HBSS containing calcium supplemented with Liberase TL Research Grade (0.5 mg/mL; Roche), hyaluronidase (0.1 mg/mL; Sigma-Aldrich), and DNase I (0.1 mg/mL; Sigma-Aldrich) for 1.5 h at 37 ºC with shaking. The digested tissue was sequentially filtered through 70-μm and 40-μm cell strainers. Red blood cells were removed using ACK lysis buffer (Gibco), and debris was depleted by Percoll (Sigma-Aldrich) density-gradient centrifugation. The resulting single-cell suspensions were used for library preparation. Single-cell 3’ gene expression libraries were generated using the Chromium GEM-X Single Cell 3’ RNA Library v4 kit (10x Genomics) according to the manufacturer’s instructions and sequenced on an Illumina platform at Macrogen (Seoul, Republic of Korea). Library preparation and sequencing were performed in a blinded manner.

### scRNA-seq data processing and cell-type annotation

Raw base call files were first converted into FASTQ format using BCL Convert (v4.3.6). Sequencing reads were then processed with Cell Ranger (v9.0.1, 10x Genomics) against the GRCm39-2024-A reference genome to obtain gene-by-cell count matrices. To exclude low-quality profiles, cells were filtered based on gene detection and mitochondrial transcript proportion, removing cells with fewer than 200 genes, more than 8,000 genes, or greater than 10% mitochondrial content. Putative doublets were identified using DoubletFinder (v2.0.4) and excluded. Following normalization, highly variable genes (top 2,000) were selected to capture transcriptional variability. Data integration across conditions and datasets was performed using an anchor-based approach implemented in Seurat (v5.0.3).

### scRNA-seq data clustering, visualization, and annotation

Cell populations were identified by constructing a shared nearest-neighbor (SNN) graph and applying community detection using the Louvain algorithm. Low-dimensional embeddings were generated using Uniform Manifold Approximation and Projection (UMAP) or t-distributed stochastic neighbor embedding (t-SNE) to visualize transcriptional heterogeneity. Cell-type identities were assigned based on canonical marker genes and prior literature 4, 27. Expression patterns and gene co-expression relationships were examined using visualization approaches such as DotPlot and FeaturePlot, with blending enabled (threshold = 0.5).

### Pseudobulk analysis and functional interpretation

To assess condition-dependent transcriptional changes, pseudobulk differential expression analysis was performed by aggregating gene counts per sample using Seurat’s pseudobulk framework. Differentially expressed genes (DEGs) were determined based on a threshold of |log2 fold change| > 1 across experimental conditions. Protein–protein interaction networks were constructed using STRINGdb (v2.16.4), and community detection was performed with the igraph R package (v2.0.3). Functional enrichment analyses were conducted using gprofiler2 (v0.2.3) and fgsea (v1.30.0). AddModuleScore function in Seurat was used for androgen receptor (AR) activity score calculation using the AR target genes obtained from the TFLink database.

### Cell–cell communication analysis

Intercellular signaling networks were reconstructed using the CellChat R package (v2.1.1), which infers ligand–receptor interactions from curated signaling databases and estimates communication strength between distinct cell populations. Since DKK signaling interactions were not included in the original CellChat database, DKK-associated ligand–receptor pairs curated from the literature were additionally incorporated for analysis (Table S1). This framework enabled the identification of key signaling sources and target cell types within the AGA microenvironment. In addition, it was applied to evaluate alterations in intercellular communication patterns in response to DHT exposure and DKK3 neutralization.

### Immunostaining

Immunofluorescence analyses were performed on formalin-fixed (10%) and paraffin-embedded sections of mouse skin and human hair follicles. Following heat-induced antigen retrieval, tissue sections were incubated with primary antibodies against DKK3 (ABclonal, Wuhan, China) and CKAP4 (Santa Cruz Biotechnology, Dallas, TX, USA). Alexa Fluor 488- or Alexa Fluor 594-conjugated secondary antibodies specific for rabbit or mouse IgG (Invitrogen, Carlsbad, CA, USA) were subsequently applied. Fluorescent images were obtained using a Nikon Eclipse Ts2 microscope (Nikon, Tokyo, Japan).

### Statistical analysis

Data are presented as mean ± standard deviation (SD) from at least three independent experiments. Comparisons between two groups were conducted using Student’s t-test. For multiple group comparisons, one-way analysis of variance (ANOVA) followed by Tukey’s post hoc test was applied. Statistical significance was defined as ^*^P < 0.05, ^**^P < 0.01 and ^***^P < 0.001. All statistical analyses were performed using GraphPad Prism 5.01 (GraphPad Software Inc., San Diego, CA, USA).

## Results

### DKK3-CKAP4 signaling is enriched in mouse and human skin

**Figure 1:**
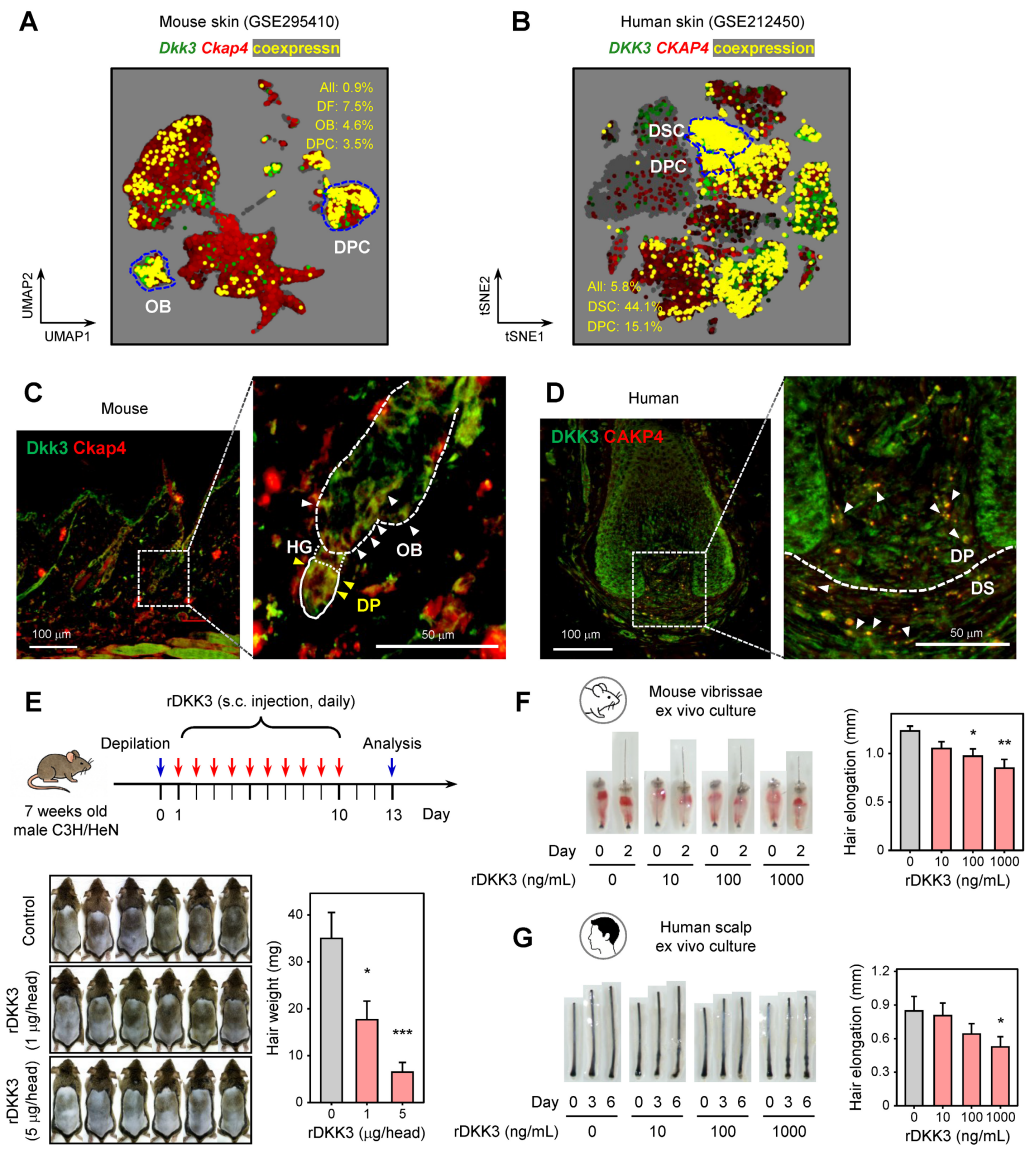
** DKK3–CKAP4 signaling is enriched in mouse and human hair follicles and recombinant DKK3 suppresses hair growth. (A, B)** UMAP embedding showing the co-expression patterns of *Dkk3* (*DKK3*) and *Ckap4* (*CKAP4*) in single-cell RNA-seq datasets of mouse dorsal skin (GSE295410, **A**) and human scalp (GSE212450, **B**). Cells expressing *Dkk3* are shown in green, *Ckap4* in red, and co-expressing cells in yellow. The percentages of co-expressing cells across all populations and within specific cell types are indicated. **(C, D)** Immunofluorescence staining of mouse (**C**) and human (**D**) HFs showing DKK3 (green) and CKAP4 (red) localization. Magnified views of the dotted boxes are shown on the right. In mouse HFs (**C**), the outer bulge (OB) is outlined with a dotted line and the dermal papilla (DP) with a solid line. Yellow arrowheads indicate co-localization in the DP and white arrowheads in the OB. In human HFs (**D**), white arrowheads indicate co-localization at the DP/dermal sheath (DS) boundary. Scale bars: left, 100 μm; right, 50 μm. **(E)** C3H/HeN mice were depilated and treated daily with rDKK3 (1 or 5 μg/head) for 10 days. Representative dorsal images on day 13 show suppressed hair regrowth in rDKK3-treated groups compared to control (PBS). Quantification of hair weight on day 13 confirmed a dose-dependent inhibition of hair growth by rDKK3 (*n* = 6 per group). ^*^P < 0.05, ^***^P < 0.001. **(F, G)** Representative images and quantitative measurements of hair shaft elongation in mouse vibrissae (**F**) and human scalp (**G**) HF organ cultures treated with rDKK3 (10, 100, 1000 ng/mL). Data are presented as mean ± s.e.m. (*n* = 10).

We first investigated the cellular context of DKK3 signaling within hair follicles. We performed a meta-analysis of publicly available single-cell RNA-seq datasets from mouse (GSE295410) and human (GSE212450) skin to map the expression of DKK family ligands and their receptors. *Dkk3* was broadly expressed across various mesenchymal lineages in mouse skin, including DFs and DPCs, as well as in certain epithelial subsets (outer bulge keratinocytes) and colocalized with *Ckap4* (Figure 1A). In human scalp, *DKK3* and *CKAP4* was robustly expressed in dermal papilla and dermal sheath clusters (Figure 1B). This co-distribution of ligand and receptor suggests the potential for local DKK3–CKAP4 signaling loops in regulating hair growth or loss.

To validate the spatial relationship between DKK3 and CKAP4 in the hair follicle, we next examined DKK3 and CKAP4 at the protein level by immunofluorescence. In mouse dorsal skin sections, Dkk3 and Ckap4 proteins showed overlapping localization in hair follicles. Dkk3 immunoreactivity was strong in the outer bulge region and in DFs surrounding the follicle, while Ckap4 immunoreactivity was seen in the dermal papilla and DFs; importantly, co-localized signals (appearing yellow) were detected in the dermal papilla and bulge area (Figure 1C). In human scalp hair follicles, DKK3 protein was abundantly expressed in the dermal papilla and dermal sheath, and CKAP4 was likewise found in these regions (Figure 1D). Merged images revealed co-localization of DKK3 and CKAP4 in the interface between the dermal papilla and sheath. These histological observations corroborate the notion that DKK3 can signal to its CKAP4 receptor within the key mesenchymal niches of the hair follicle (dermal papilla/sheath).

### Recombinant DKK3 suppresses hair growth

To evaluate the effect of DKK3 on hair growth, we first employed a depilation-induced hair regeneration model in C3H/HeN mice. Subcutaneous administration of recombinant DKK3 (rDKK3) to telogen-phase mouse skin over 10 days markedly delayed the onset of hair regrowth. By day 13 post-depilation, rDKK3-treated mice showed visibly reduced new hair coverage and significantly lower hair weight compared to PBS-treated controls (Figure 1E), indicating an inhibition of telogen-to-anagen transition. Consistently, in *ex vivo* organ cultures, rDKK3 suppressed hair shaft elongation in both mouse vibrissae follicles (Figure 1F), and human scalp hair follicles in a dose-dependent manner (Figure 1G). These data suggest that DKK3 appears to function as a negative regulator of hair follicle growth in both species.

### DKK3 neutralization promotes hair growth

**Figure 2:**
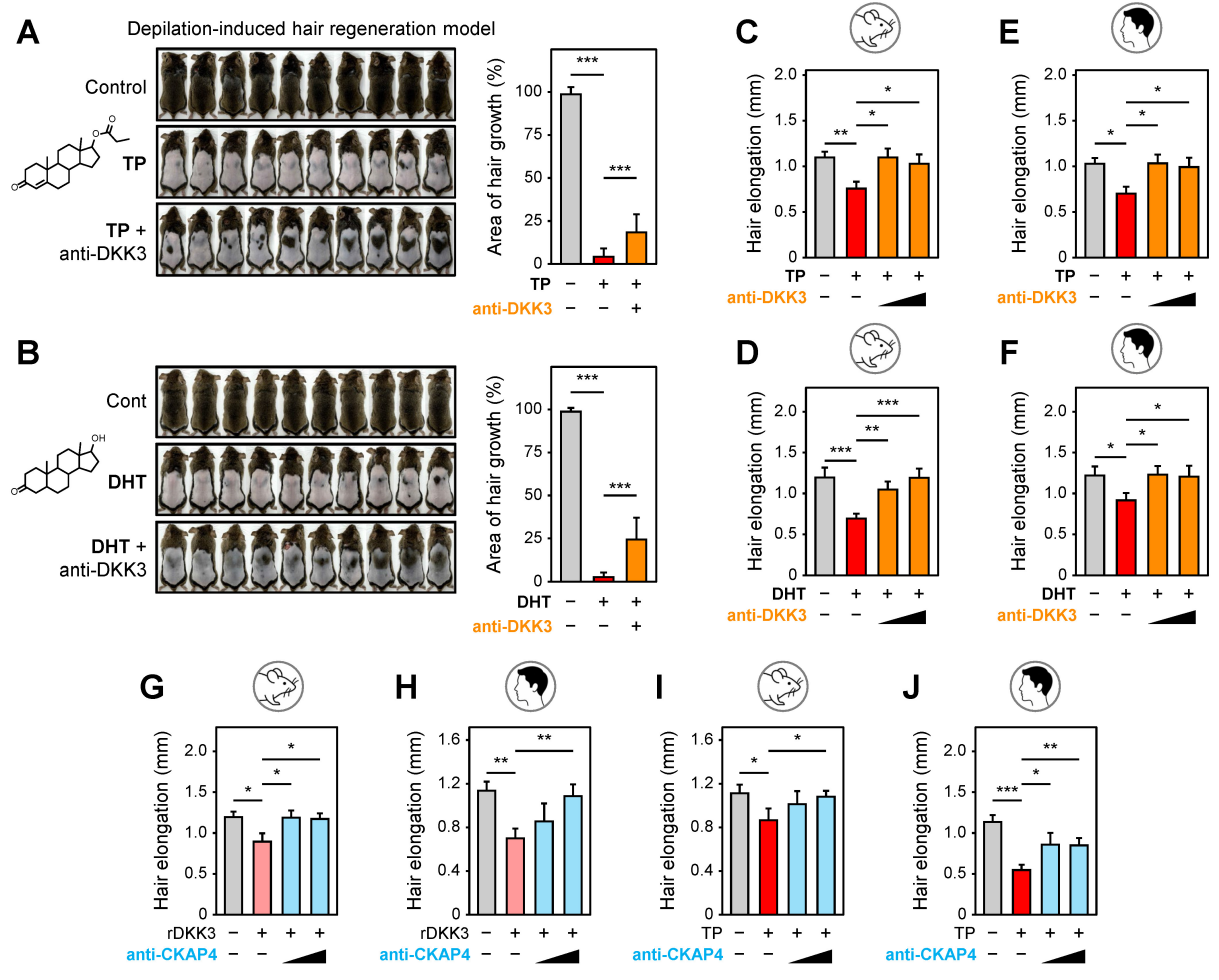
** Neutralization of DKK3 and CKAP4 promotes hair growth in androgen-induced hair loss models. (A, B)** Representative dorsal photographs and quantification of hair regrowth area (%, mean ± s.e.m.) on day 19 in mice treated with testosterone propionate (TP, **A**) or dihydrotestosterone (DHT, **B**) with or without anti-DKK3 antibody. ^***^P < 0.001. **(C, D)** In mouse vibrissae hair follicle organ cultures, TP (100 nM) or DHT (100 nM) suppressed hair shaft elongation, which was reversed by anti-DKK3 antibody (10 and 100 ng/ml). ^*^P < 0.05, ^**^P < 0.01, ^***^P < 0.001. **(E, F)** In human hair follicle organ cultures, TP (100 nM) or DHT (100 nM) suppressed hair shaft elongation, which was rescued by anti-DKK3 antibody treatment (100 and 1000 ng/ml). ^*^P < 0.05. **(G)** Mouse vibrissae follicle organ culture treated with rDKK3 (1 μg/mL) in the presence or absence of anti-CKAP4 antibody (10 and 100 ng/mL). ^*^P < 0.05. **(H)** Human hair follicle organ culture treated with rDKK3 (1 μg/ml) in the presence or absence of anti-CKAP4 antibody (0.1 and 1 μg/mL). ^**^P < 0.01.** (I)** Mouse vibrissae follicle organ culture treated with TP (100 nM) in the presence or absence of anti-CKAP4 antibody (10 and 100 ng/mL). ^*^P < 0.05. **(J)** Human hair follicle organ culture treated with TP (100 nM) in the presence or absence of anti-CKAP4 antibody (0.1 and 1 μg/mL). ^*^P < 0.05, ^**^P < 0.01, ^***^P < 0.001.

As rDKK3 inhibits hair growth, blocking DKK3 in an androgen-rich environment might alleviate hair loss. To test this, we treated C3H mice with TP or DHT to induce AGA-like hair growth inhibition, and simultaneously administered a neutralizing anti-DKK3 monoclonal antibody (mAb) or control. *In vivo*, both TP and DHT treatment led to significant hair growth inhibition, as evidenced by delayed skin re-pigmentation (anagen entry) and poor regrowth of hair on the mice’s dorsum (Figure 2A-B, left panels). Quantification of hair re-growth area confirmed that neutralizing DKK3 rescued hair regrowth despite the continued presence of high androgen (Figure 2A-B, right graphs). Similarly, in organ culture models, we found that anti-DKK3 mAb could reverse the hair growth suppression caused by androgens. Mouse vibrissae follicles treated with TP or DHT had significantly shorter hair shafts than controls, but treatment of anti-DKK3 (at 10–100 ng/mL) restored hair shaft elongation in a dose-dependent manner (Figure 2C-D). Human hair follicles treated with 100 nM TP or DHT *ex vivo* exhibited minimal growth over 6 days, whereas those co-treated with an anti-DKK3 antibody (100–1000 ng/mL) showed a significant rescue of hair elongation (Figure 2E-F). These data indicate that neutralizing DKK3 partially restores the regenerative capacity of hair follicles even under ongoing androgenic stimulation.

### CKAP4 neutralization alleviates DKK3- or androgen-induced hair regression

We then tested whether blocking CKAP4 could mitigate the hair-inhibitory effects of DKK3 and/or androgen. In initial organ culture experiments, we added a neutralizing anti-CKAP4 antibody in the presence of exogenous rDKK3. In both mouse vibrissa and human hair follicle cultures, anti-CKAP4 significantly rescued hair shaft elongation that was suppressed by rDKK3 (Figure 2G-H). This confirms that CKAP4 is indeed the functionally relevant receptor through which DKK3 impairs hair growth; blocking CKAP4 abrogates DKK3’s action.

Next, we assessed whether CKAP4 is also required for androgen-induced hair growth suppression. In TP-treated organ cultures, addition of anti-CKAP4 antibody led to notable improvements in hair follicle growth. Mouse vibrissae follicles grown with TP plus anti-CKAP4 showed longer hair shafts than those with TP alone, effectively neutralizing the TP effect (Figure 2I). Likewise, human hair follicles treated with TP in the presence of anti-CKAP4 exhibited significantly greater elongation than TP-only follicles (Figure 2J). These results indicate that CKAP4 appears to mediate, at least in part, not only DKK3-driven, but also androgen-driven, hair growth inhibition, implicating CKAP4-dependent signaling (likely via endogenous DKK3) in the AGA mechanism.

### scRNA-seq analysis reveals cellular and molecular mechanisms underlying DKK3-mediated androgenic hair follicle degeneration

**Figure 3:**
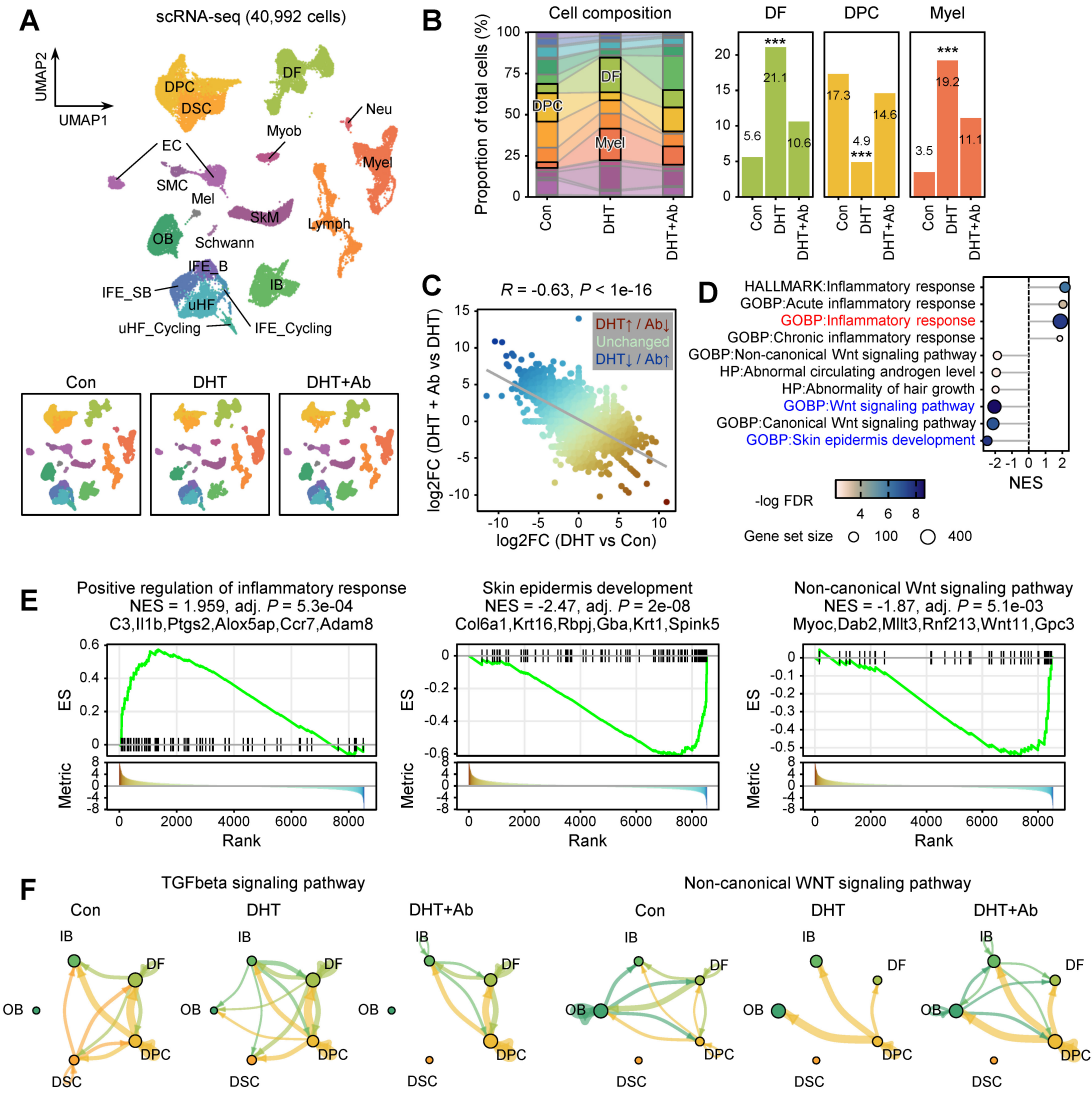
** Single-cell transcriptomic analysis reveals DKK3-dependent remodeling of the follicular microenvironment in DHT-induced AGA model. (A)** UMAP visualization of 40,992 single cells obtained from dorsal skin of control (Con), DHT-induced AGA (DHT), and DHT-treated skin co-administered with an anti-DKK3 antibody (DHT+Ab). Cells were annotated into 19 major lineages, including: interfollicular epidermal keratinocytes (IFE_B, basal; IFE_SB, suprabasal; IFE_Cycling), upper hair follicle keratinocytes (uHF, uHF_Cycling), outer bulge (OB) and inner bulge (IB) hair follicle keratinocytes, mesenchymal populations (dermal fibroblasts [DF], dermal papilla cells [DPC], dermal sheath cells [DSC]), immune cells (lymphocytes [Lymph], myeloid cells [Myel], neutrophils [Neu]), and other cell types including myoblasts (Myob), skeletal muscle (SkM), smooth muscle cells (SMC), endothelial cells (EC), Schwann cells, and melanocytes (Mel). **(B)** Changes in cell-type composition across experimental conditions. Statistical significance was determined by a binomial test (^***^P < 0.001). **(C)** Scatter plot correlating transcriptional changes between [DHT vs. Con] and [DHT+Ab vs. DHT]. Each dot represents an individual gene, color-coded by the difference in fold change (log_2_FC [DHT vs. Con] – log_2_FC [DHT+Ab vs. DHT]). **(D)** Gene set enrichment analysis (GSEA) of pseudobulk differentially expressed genes (DEGs) using *fgsea*. The input was ranked by the difference in fold change against Gene Ontology (GO) terms. **(E)** Representative GSEA plots for “Positive regulation of inflammatory response,” “Skin epidermis development,” and “Non-canonical Wnt signaling pathway.” Normalized enrichment score (NES) and leading-edge genes are indicated. **(F)** CellChat analysis illustrating TGF-β and non-canonical WNT signaling pathways across HF keratinocytes and mesenchymal cells. The thickness of lines connecting cell types is proportional to the calculated interaction strength.

To elucidate the cellular mechanisms underlying the phenotypic rescue observed upon DKK3 neutralization, we performed scRNA-seq of dorsal skin from three experimental conditions: control (Con), DHT-induced AGA, and DHT-treated skin co-administered with an anti-DKK3 antibody (DHT+Ab). After quality control and intensive manual curation, we obtained transcriptomic profiles from 40,992 single cells, which were classified into 19 distinct cell lineages. These included basal and suprabasal interfollicular epidermal keratinocytes (IFE_B and IFE_SB), upper hair follicle (uHF) keratinocytes, outer bulge (OB) and inner bulge (IB) hair follicle keratinocytes, mesenchymal lineages (DFs, DPCs, DSCs), immune populations (lymphocytes, myeloid cells, neutrophils), as well as muscle and endothelial cells (Figure 3A, Figure S2).

Comparison of cell-type composition across conditions revealed pronounced androgen-induced remodeling of the follicular microenvironment. Notably, mesenchymal and immune populations expanded markedly upon DHT treatment. DFs increased from 5.6% of total cells in control skin to 21.1% in the DHT condition (Figure 3B). In contrast, the proportion of DPCs was substantially reduced under DHT exposure. Importantly, anti-DKK3 antibody treatment partially reversed these changes: DF cell fractions were reduced to 10.6%, while DPC representation was restored toward control levels (Figure 3B).

To characterize the transcriptional programs underlying this reversal, we performed pseudobulk differential expression analysis across conditions (Figure 3C). A correlation analysis between the fold changes revealed a significant inverse correlation (Pearson *R* = -0.63), demonstrating that a substantial proportion of DHT-induced gene expression changes were partially reversed by anti-DKK3 antibody treatment. Gene set enrichment analysis (GSEA) further showed that DHT treatment induced robust upregulation of inflammatory and profibrotic gene programs, whereas anti-DKK3 treatment attenuated these signatures (Figure 3D-E, Figure S3). On the contrary, DHT treatment reduced the Wnt signaling pathway and skin epidermis development, whereas anti-DKK3 treatment attenuated these signatures (Figure 3D-E). Furthermore, CellChat-based cell-cell communication analysis revealed that DHT treatment markedly enhanced TGF-β signaling between mesenchymal populations (including DFs and DPCs) and epithelial populations (including OB keratinocytes); however, this signaling was weakened upon DKK3 inhibition (Figure 3F). In parallel, the strength of non-canonical Wnt signaling, which was diminished by DHT, showed a clear pattern of recovery following anti-DKK3 treatment (Figure 3F).

### Androgen-driven AR activity activates fibroimmune transcriptional programs in DPCs

**Figure 4:**
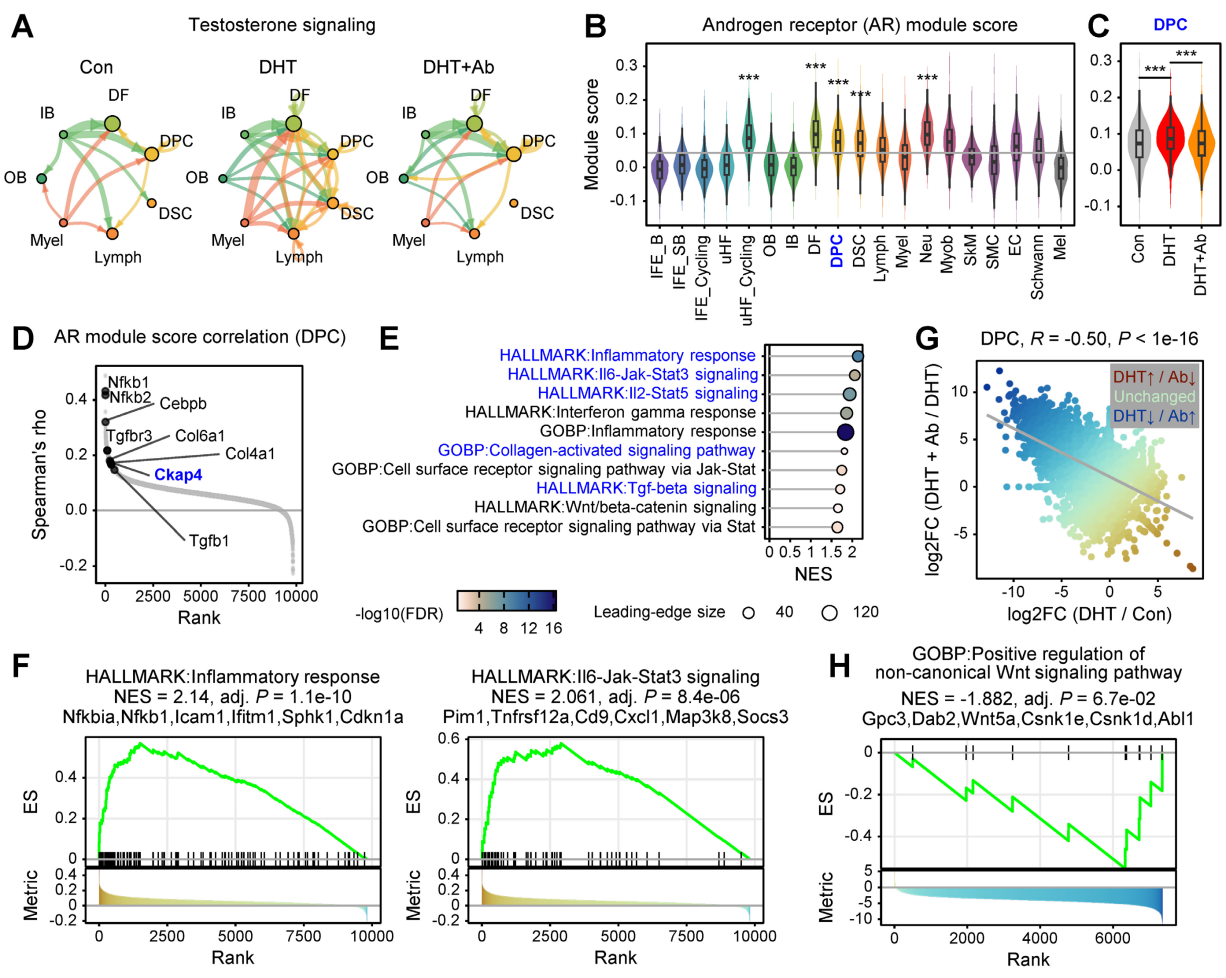
** Androgen-driven AR activity activates fibroimmune transcriptional programs in dermal papilla cells. (A)** CellChat analysis showing enhanced testosterone-related signaling interactions within the hair follicle niche. Line thickness represents interaction strength. **(B)** Androgen receptor (AR) target gene module scores across cell populations were calculated using *AddModuleScore* function in Seurat. Scores were compared using a two-sided t-test (^***^P < 0.001). **(C)** Violin plots showing AR module scores in DPCs across conditions. **(D)** Rank-order plot of genes correlated with AR module scores in DPCs. Dots represent individual genes ranked by Spearman’s rho. **(E, F)** GSEA of AR-correlated genes in DPCs. Dot size represents the number of genes in the leading edge; color represents -log_10_(FDR), and representative GSEA plots are shown (**F**). **(G)** Scatter plot correlating transcriptional changes in DPCs between [DHT vs. Con] and [DHT+Ab vs. DHT]. **(H)** GSEA of DEGs in DPCs ranked by the difference in fold change against a GO term, “Positive regulation of non-canonical Wnt signaling pathway.”

CellChat analysis revealed that DHT treatment enhances androgen-related intercellular signaling within the hair follicle microenvironment, characterized by increased interaction strength and connectivity among mesenchymal and epithelial cell populations (Figure 4A). In contrast, DKK3 inhibition suppresses DHT-induced androgen signaling networks, thereby modulating androgen-driven cellular crosstalk within the follicular niche. Next, we quantified AR activity at the single-cell level by calculating AR target gene module scores. Consistent with prior observations 4, AR activity was highest in mesenchymal compartments, including DFs, DPCs, and DSCs (Figure 4B). Notably, AR module scores in DPCs were significantly elevated under DHT treatment and were normalized upon anti-DKK3 antibody administration (Figure 4C), suggesting that DKK3 contributes to sustained androgen-responsive transcriptional states in mesenchyme. To further dissect androgen-associated transcriptional changes, we examined genes whose expression correlated with AR module scores in DPCs. This analysis identified strong positive correlations with multiple collagen genes (*Col3a1*, *Col4a1*, *Col6a1*) as well as inflammatory transcriptional regulators such as *Ckap4*, *Cebpb* and *Nfkb1* (Figure 4D). GSEA showed that AR activation was associated with increased enrichment of several signaling pathways, including inflammatory response, JAK–STAT3 signaling, IL2–STAT5 signaling, TGF-β signaling, and Wnt/β-catenin signaling (Figure 4E-F, Figure S4).

We then examined the correlation between gene expression fold changes within the DPC population (Figure 4G). This revealed a significant inverse correlation (Pearson *R* = -0.5), confirming that DKK3 neutralization effectively reverses the majority of androgen-induced transcriptomic shifts within DPCs. Notably, GSEA of genes whose expression was induced by DHT but attenuated by anti-DKK3 treatment highlighted a significant association with the non-canonical Wnt signaling pathway within DPCs (Figure 4H). Collectively, these results suggest that AR activation promotes inflammatory and fibrotic programs in DPCs while simultaneously disrupting key developmental pathways, emphasizing that the therapeutic efficacy of DKK3 inhibition is closely linked to the restoration of these developmental cues, particularly the non-canonical Wnt signaling axis.

To validate whether the fibroimmune transcriptional programs predicted by scRNA-seq and CellChat analyses are manifested at the tissue level, we performed immunostaining analyses in androgen-induced AGA mouse skin. Immunostaining revealed that DHT treatment markedly increased MHC-I expression in the dermal compartment, indicating enhanced immune activation within the skin microenvironment. In parallel, macrophage infiltration, as assessed by F4/80 staining, was significantly elevated in DHT-treated skin, consistent with the increased chemokine signaling and immune cell recruitment predicted by our transcriptomic analysis. Furthermore, TGF-β1 expression was prominently induced following androgen exposure, suggesting activation of extracellular matrix (ECM) remodeling and fibroblast activation associated with perifollicular fibrosis. Importantly, these fibroimmune features were attenuated upon treatment with a DKK3-neutralizing antibody. Specifically, anti-DKK3 treatment reduced MHC-I expression, decreased F4/80⁺ macrophage infiltration, and suppressed TGF-β1 levels in the dermal region. These findings provide direct *in vivo* evidence that androgen-driven fibroimmune remodeling occurs at the tissue level and that DKK3 blockade mitigates both immune activation and fibrotic responses within the hair follicle microenvironment (Figure S5).

### DKK3 secreted from outer bulge activates CKAP4 in DPCs

**Figure 5:**
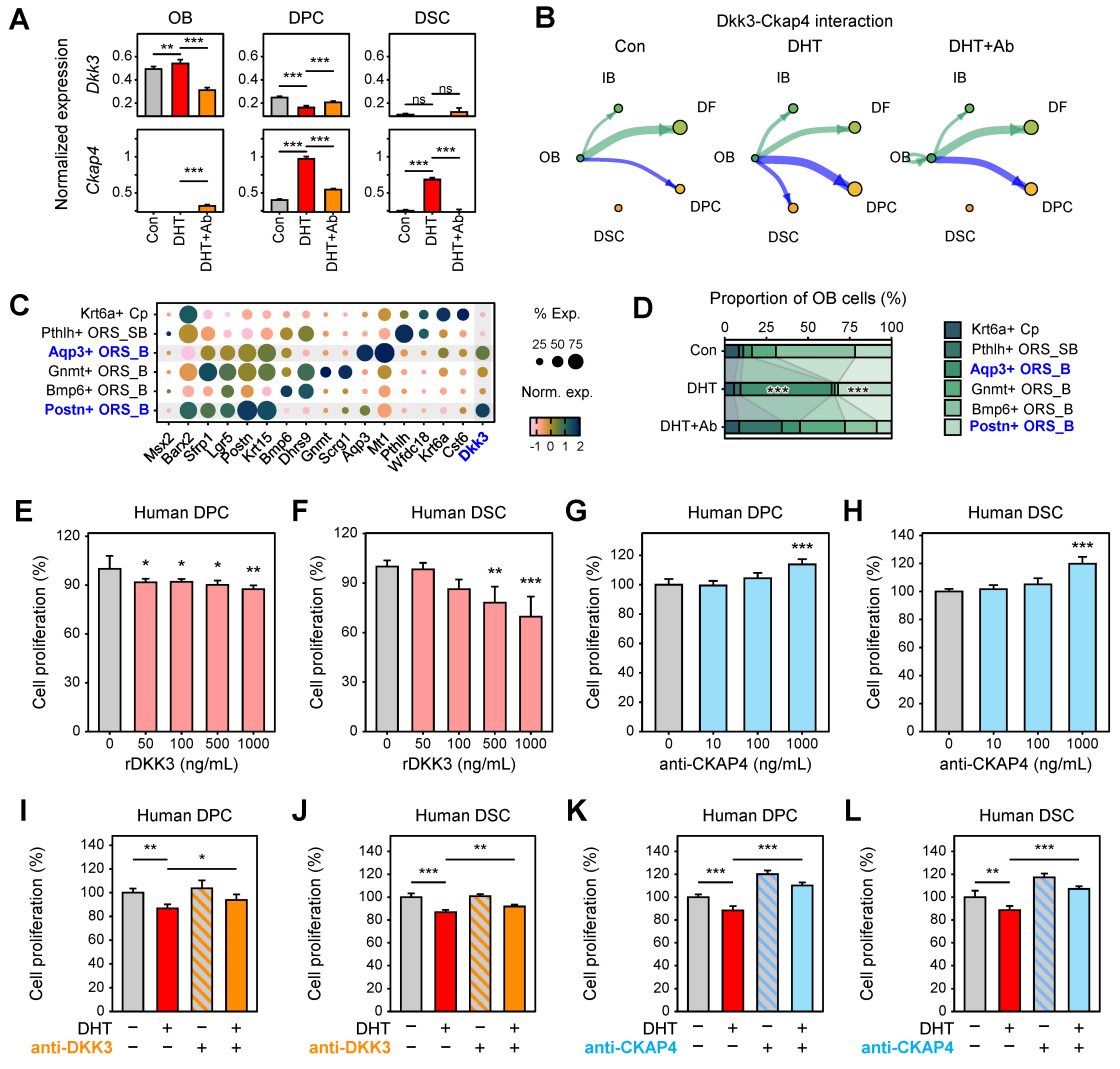
** DKK3 secreted from outer bulge keratinocytes activates CKAP4 signaling in dermal papilla and dermal sheath cells. (A)** Expression levels of *Dkk3* and *Ckap4* across experimental conditions (Wilcoxon test, ^**^P < 0.01, ^***^P < 0.001). **(B)** CellChat analysis of Dkk3–Ckap4 interaction across HF keratinocytes and mesenchymal compartments.** (C)** Dot plot showing marker gene expression defining OB keratinocyte subpopulations, including outer root sheath (ORS) and companion layer (Cp) cells: Postn+ ORS_B (ORS basal), Bmp6+ ORS_B, Gnmt+ ORS_B, Aqp3+ ORS_B, Pthlh+ ORS_SB (ORS suprabasal), and Krt6a+ Cp cells. **(D)** Compositional changes of OB subpopulations across conditions (left) and their distribution. Statistical significance was determined by a binomial test (^***^P < 0.001). **(E, F)** Effects of rDKK3 on proliferation of cultured human DPCs (**E**) and DSCs (**F**), showing dose-dependent suppression of cell proliferation. ^*^P < 0.05, ^**^P < 0.01, ^***^P < 0.001. **(G, H)** Effects of CKAP4 inhibition on the proliferation of DPCs (**G**) and DSCs (**H**), demonstrating restoration of cell proliferation following CKAP4 blockade. **(I, J)** Effects of anti-DKK3 antibody on DHT-induced suppression of cell proliferation in DPCs (**I**) and DSCs (**J**). ^*^P < 0.05, ^**^P < 0.01, ^***^P < 0.001. **(K, L)** Effects of CKAP4 inhibition on DHT-induced suppression of cell proliferation in DPCs (**K**) and DSCs (**L**). ^**^P < 0.01, ^***^P < 0.001.

Analysis of ligand and receptor expression patterns revealed spatially distinct regulation of the DKK3–CKAP4 axis. *Dkk3* expression was primarily localized to OB keratinocytes; its expression was markedly induced under DHT treatment and subsequently reduced upon anti-DKK3 antibody administration. In contrast, *Ckap4* expression was enriched in mesenchymal compartments, particularly in DPCs and DSCs, and exhibited similar expression dynamics following DHT exposure and antibody treatment (Figure 5A). CellChat analysis further demonstrated that Dkk3–Ckap4 signaling was increased from OB keratinocytes to DPCs and DSCs following DHT treatment, whereas this interaction was markedly suppressed by treatment with a DKK3-neutralizing antibody, indicating that DHT enhances DKK3-mediated stromal signaling within the follicular niche (Figure 5B). OB keratinocytes consist of diverse subpopulations, including Postn^+^ basal ORS (ORS_B), Bmp6^+^ ORS_B, Gnmt^+^ ORS_B, Aqp3^+^ ORS_B, Pthlh⁺ suprabasal ORS (ORS_SB), and Krt6a^+^ companion layer (Cp) cells (Figure 5C). Interestingly, while most OB subpopulations significantly decreased in DHT-treated skin, the Dkk3-expressing populations, specifically Postn^+^ ORS_B and Aqp3^+^ ORS_B cells, were markedly expanded. These populations were subsequently reduced following anti-DKK3 antibody treatment (Figure 5D), suggesting that the expansion of these specific OB subsets primarily drives the elevated levels of OB-derived DKK3 in AGA. We further detected the increased protein expression of AQP3 in DHT mouse, which was reduced by DKK3 antibody treatment (Figure S6).

To experimentally validate these findings and assess their functional relevance in human models, we examined the effects of rDKK3 and CKAP4 antibody on the proliferation of DPCs and DSCs. In cultured human cells, rDKK3 significantly reduced the proliferation of DPCs (Figure 5E) and DSCs (Figure 5F) in a dose-dependent manner. In contrast, inhibition of CKAP4 significantly induced the proliferation of DPCs (Figure 5G) and DSCs (Figure 5H) in a dose-dependent manner. Collectively, these findings indicate that DKK3 secreted from OB keratinocytes activates CKAP4 signaling in DPCs and DSCs, thereby promoting stromal signaling changes that contribute to hair follicle miniaturization in AGA.

To further evaluate the functional relevance of the DKK3–CKAP4 axis under androgenic conditions, we next examined whether inhibition of this pathway could reverse DHT-induced suppression of cell proliferation. Consistent with previous observations, DHT treatment significantly reduced the proliferation of both DPCs and DSCs. Notably, inhibition of DKK3 significantly reversed DHT-induced suppression, leading to a recovery of proliferative capacity in both cell types (Figure 5I-J). Similarly, treatment with an anti-CKAP4 antibody restored cell proliferation under DHT conditions (Figure 5K-L). These results demonstrate that blockade of the DKK3–CKAP4 signaling axis effectively counteracts androgen-mediated growth inhibition in dermal papilla and dermal sheath cells, providing direct functional evidence supporting its role in AGA pathogenesis.

### Androgen-driven AR activity activates fibroimmune transcriptional programs in dermal fibroblasts

**Figure 6:**
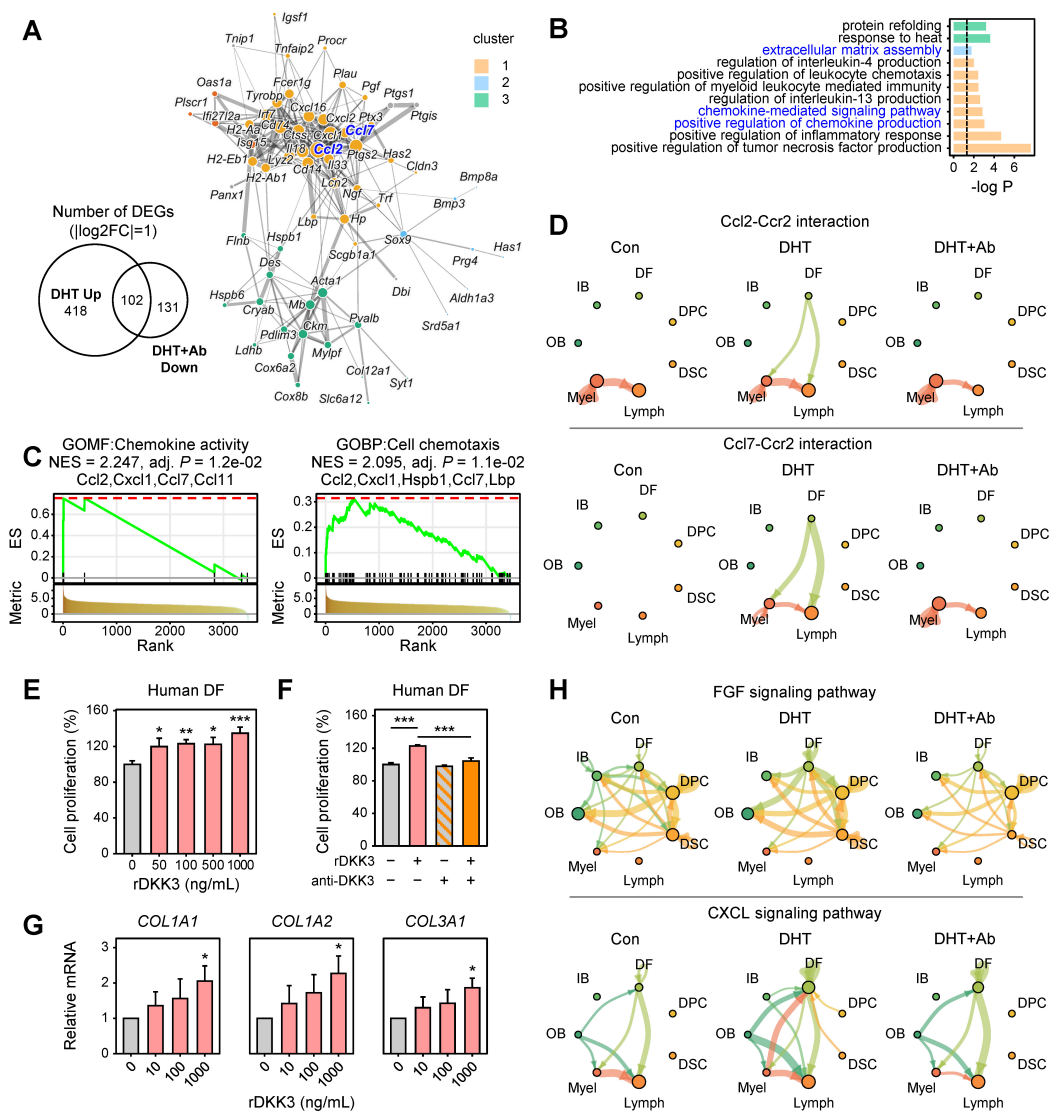
** DKK3 promotes fibroimmune activation and perifollicular fibrosis in dermal fibroblasts. (A)** STRING protein–protein interaction (PPI) network of genes upregulated by DHT and downregulated by anti-DKK3 antibody in DFs. Clusters were identified using the edge-betweenness (Girvan–Newman) algorithm. **(B)** GO enrichment analysis of the three identified PPI clusters. **(C)** GSEA of DEGs in DFs, highlighting chemokine activity and cell chemotaxis pathways. **(D)** CellChat analysis of chemokine signaling (Ccl2–Ccr2 and Ccl7–Ccr2 interactions) originating from DFs toward immune populations. **(E)** Effects of rDKK3 on proliferation of cultured human DFs. ^*^P < 0.05, ^**^P < 0.01, ^***^P < 0.001. **(F)** Neutralization of DKK3 suppresses rDKK3-induced DF proliferation. **(G)** qPCR analysis showing upregulation of fibrosis-associated genes, including *COL1A1*, *COL1A2*, and *COL3A1*, following rDKK3 treatment. **(H)** CellChat analysis of intercellular communication networks for the FGF and CXCL12 signaling pathways originating from DFs.

Given that DFs exhibited high AR module scores and were identified as one of primary targets of AR signaling (Figure 4B), we further investigated the transcriptional changes in this population. We identified 102 differentially expressed genes (DEGs) in DFs that were significantly upregulated by DHT and effectively reversed upon DKK3 neutralization (Figure 6A). Protein–protein interaction (PPI) network analysis using STRING revealed that these DEGs formed three highly interconnected clusters. Gene Ontology (GO) enrichment analysis of these clusters showed that they were primarily associated with ECM organization and chemokine-mediated signaling pathways (Figure 6B). GSEA further confirmed that genes related to chemokine activity and chemotaxis, including *Ccl2*, *Cxcl1*, *Ccl7*, *Ccl11*, *Hspb1*, and *Lbp*, were significantly upregulated under DHT treatment but attenuated by anti-DKK3 (Figure 6C). These results suggest that DHT induces a pro-fibrotic and inflammatory transcriptional program in DFs, which is partially reversed by DKK3 neutralization. Consistently, CellChat analysis indicated that DFs act as a major source of chemokine signaling directed toward immune cell populations, mainly through Ccl2-Ccr2 and Ccl7-Ccr2 interactions, suggesting enhanced fibroblast-immune cell communication within the follicular niche (Figure 6D). These findings indicate that activated DFs may promote immune cell recruitment through increased chemokine signaling in the AGA microenvironment.

As DFs are abundant in the scalp mesoderm and play a pivotal role in perifollicular fibrosis, we examined the functional impact of DKK3 on these cells. Interestingly, rDKK3 exerted an effect on DFs opposite to that observed in DPCs/DSCs, significantly increasing human DF proliferation in a dose-dependent manner (Figure 6E). This pro-proliferative effect on DFs was specifically mediated by DKK3, as co-treatment with an anti-DKK3 neutralizing antibody normalized DF proliferation back to control levels (Figure 6F). In parallel, rDKK3 markedly induced fibrosis-associated genes in DFs; qPCR revealed upregulation of ECM components *COL1A1*, *COL1A2*, and *COL3A1* (type I and III collagen isoforms; Figure 6G). Consistently, CellChat analysis revealed enhanced FGF and CXCL12 signaling originating from DFs, which are known to regulate fibroblast proliferation and ECM production (Figure 6H). Collectively, these results suggest that activated DFs may contribute to perifollicular fibrotic remodeling through increased growth factor and chemokine-mediated signaling within the follicular microenvironment.

## Discussion

Recent single-cell transcriptomic analyses revealed a striking divergence in the expression patterns of DKK family members and their cognate receptors between mouse (GSE295410) and human skin (GSE212450). As shown in Figure S1, human DPCs and DSCs exhibited strong co-expression of *DKK3* and its noncanonical receptor *CKAP4*, whereas the expression of *DKK1* and its canonical Wnt receptors *LRP5/6* was notably weak in the same cell types. In contrast, mouse skin showed predominant expression of *Dkk2*-*Lrp6* and *Dkk2*-*Kremen1/2* interactions, with relatively lower contribution from *Dkk3*-*Ckap4* signaling. This species-specific divergence underscores the importance of validating Wnt modulator function and therapeutic targets in human-derived models 29, 30. While DKK1 has been widely studied in mouse models of HF cycling and regression, our data suggest that DKK3 plays a more central role in the human HF microenvironment, particularly under androgenic stimulation. These findings caution against direct extrapolation from murine models and highlight the necessity of human single-cell-resolved approaches in dissecting the pathological signaling landscape of AGA.

Although members of the DKK family share structural homology, their expression profiles and functional consequences in the HF microenvironment appear to be distinct 30, 31. In our study and prior transcriptomic datasets (e.g., GSE295410), DKK1 mRNA was found to be weakly expressed in DPCs and ORS cells, despite its well-known upregulation by DHT 8. In a preliminary study, we found that DKK1 antibody promoted hair growth in human hair organ culture and accelerate telogen-to-anagen transition in AGA mouse (Figure S7). DKK1 transcripts were predominantly detected in adipose-derived stem cells (ASCs) located around the HF 8, suggesting that Dkk1 derived from ASCs might be involved in hair loss in AGA. Conversely, DKK2, another canonical Wnt antagonist, has been reported to exert hair growth-promoting effects 32. Prior *in vivo* studies have demonstrated that recombinant DKK2 protein can stimulate anagen entry and enhance hair shaft elongation, in contrast to the hair-inhibitory actions of DKK1 32. Notably, DKK3 expression is highly enriched in OB keratinocytes, and its signaling through CKAP4 correlates with androgen-induced hair follicle degeneration. In both human scalp tissue and mouse models, recombinant DKK3 treatment led to significant suppression of hair elongation, while DKK3 neutralizing antibodies rescued hair growth in testosterone- or DHT-induced alopecia settings. These findings underscore that, unlike DKK1 or DKK2, DKK3 may act as an important contributor to hair follicle miniaturization in AGA. Functional differences between DKK family members further support this divergence. While DKK1 has been primarily associated with hair cycle inhibition via suppression of Wnt-dependent epithelial proliferation 10, our data demonstrate that DKK3 drives fibroimmune remodeling, including activation of TGF-β signaling, ECM deposition, and chemokine-mediated immune cell recruitment. These effects are not typically attributed to DKK1 or DKK2 and suggest that DKK3 operates through a mechanistically distinct pathway that contributes to the chronic progression of AGA.

Although both DKK1 and DKK3 have been reported to interact with the membrane receptor CKAP4, accumulating evidence suggests that the nature and functional consequences of these interactions differ significantly. DKK1 is primarily known as a canonical Wnt pathway antagonist, exerting its effects by binding to LRP5/6 and Kremen1/2 to block Wnt–Frizzled complex formation 20, 31. While recent reports have shown that palmitoylation of CKAP4 may allow DKK1 to also associate with CKAP4 at specific membrane microdomains, this interaction appears to be context-dependent and auxiliary, possibly facilitating noncanonical or redundant signaling in certain cancer models or fibroblast systems 20, 33. In contrast, DKK3 is now recognized as a noncanonical ligand for CKAP4, functioning independently of LRP5/6. This distinction is critical, as it places DKK3 outside the classical Wnt inhibitory framework that has been extensively studied in hair biology. Several studies, demonstrate that DKK3-CKAP4 binding initiates distinct downstream cascades, including Akt and NF-κB activation, without engaging β-catenin signaling 19, 34. This makes DKK3-CKAP4 interaction particularly relevant in fibrotic and inflammatory settings, where canonical Wnt modulation may be minimal, but noncanonical cues drive pathological changes. Indeed, our results indicate that DKK3-CKAP4 signaling is dominant in DPCs and DFs of the human scalp, whereas DKK1-CKAP4 contribution appears negligible in the same cell types. These differences highlight that, despite potential overlap in receptor usage, DKK1 and DKK3 engage CKAP4 through distinct signaling routes, resulting in divergent biological outcomes. For therapeutic purposes, this distinction supports the selective targeting of DKK3-CKAP4 as a disease-specific axis in AGA, without interfering with DKK1-mediated canonical Wnt regulation which may serve homeostatic or regenerative functions in other tissues 35, 36.

Given the complexity of AGA pathogenesis, which involves androgen signaling, immune activation, and ECM remodeling, it is unlikely that a single pathway fully accounts for disease progression. Instead, our data support a model in which the DKK3–CKAP4 axis acts as a context-dependent amplifier of fibroimmune remodeling downstream of androgen signaling. DKK3 secreted from OB keratinocytes binds to CKAP4 on neighboring mesenchymal cells, triggering downstream Akt and NF-κB pathways and promoting perifollicular fibrosis, immune cell infiltration, and eventual follicular miniaturization. Our findings suggest that therapeutic interruption of this axis may restore a regenerative environment and reverse the progression of AGA. Among possible strategies to block this pathway, direct neutralization of DKK3 with a monoclonal antibody offers several advantages over CKAP4-targeted approaches. As a secreted ligand, DKK3 is readily accessible to extracellular antibodies and can be selectively blocked without interfering with the broader intracellular functions of CKAP4, which serves as a scaffolding and trafficking protein in diverse physiological contexts. Therefore, anti-DKK3 monoclonal antibodies represent a promising class of targeted biotherapeutics for AGA, offering specificity, safety, and functional reversal of DKK3-driven stromal dysfunction. Our data provide a mechanistic rationale for their development as novel regenerative treatments in hair loss.

## Supplementary Material

Supplementary figures and table.

## Data Availability

The scRNA-seq data generated in this study have been deposited in the Gene Expression Omnibus (GEO) under accession number GSE325671. Publicly available datasets utilized for meta-analysis were obtained from GEO, including GSE295410 (mouse) and GSE212450 (human).

## Abbreviations

AGA: Androgenetic alopecia
AR: androgen receptor
CKAP4: cytoskeleton-associated protein 4
DEGs: differentially expressed genes
DFs: dermal fibroblasts
DHT: dihydrotestosterone
DKK: Dickkopf
DPCs: dermal papilla cells
DSCs: dermal sheath cells
ECM: extracellular matrix
GO: gene ontology
GSEA: gene set enrichment analysis
HF: hair follicle
LRP5/6: lipoprotein receptor-related protein 5/6
mAb: monoclonal antibody
OB: outer bulge
rDKK3: recombinant DKK3 protein
PPI: Protein–protein interaction
scRNA-seq: single-cell RNA sequencing
SE: standard error
TP: testosterone propionate
t-SNE: t-distributed stochastic neighbor embedding
UMAP: uniform manifold approximation and projection
