# Supplementary material for: DKK3–CKAP4 signaling drives fibroimmune remodeling and hair follicle miniaturization in androgenetic alopecia
Source: Theranostics. 2026 May 29;16(13):7124–38. doi: 10.7150/thno.135412 (PMC13294971; doi:10.7150/thno.135412)
Supplement: Supplementary file 1 — Supplementary figures and table. [file thnov16p7124s1.pdf]

DKK3–CKAP4 signaling drives fibroimmune remodeling and hair follicle miniaturization in androgenetic alopecia

Seungchan An[1,2,3,#], Hyunju Kim[1,#], Mei Zheng[1], In Guk Park[2], Leegu Song[1], Jino Kim[4], Minsoo Noh[2,*], and Jong-Hyuk Sung[1,*]

[1] Epi Biotech Co., Ltd., Incheon 21984, Republic of Korea

[2] Natural Products Research Institute, College of Pharmacy, Seoul National University, Seoul 08826, Republic of Korea

[3] Ludwig Institute for Cancer Research, Princeton University, Princeton, NJ 08544, USA

[4] New Hair Plastic Surgery Clinic, Seoul, Republic of Korea

[#]Equal contribution

∗Co-correspondence to: Jong-Hyuk Sung; email: brian99@epibiotech.com or Minsoo Noh; email: minsoonoh@snu.ac.kr

Supplementary Table 1

Ligand	Receptor	Reference
DKK1	LRP5	PMID:17143291
DKK1	LRP6	PMID:11357136
DKK1	KREMEN1	PMID:12050670
DKK1	KREMEN2	PMID:12050670
DKK1	CKAP4	PMID:27322059
DKK2	LRP5	PMID:17143291
DKK2	LRP6	PMID:11357136
DKK2	KREMEN1	PMID:17143291
DKK2	KREMEN2	PMID:12527209
DKK3	KREMEN1	PMID:20370576
DKK3	KREMEN2	PMID:20370576
DKK3	CKAP4	PMID:35708905
DKK3	ACKR3	PMID:29980568

Table S1. Literature-curated list of DKK family ligands and their cognate receptors. The table summarizes previously reported interactions between DKK1, DKK2, and DKK3 and their respective receptors, including LRP5/6, KREMEN1/2, CKAP4, and ACKR3, along with their corresponding PubMed identifiers (PMID).

Supplementary Figure 1

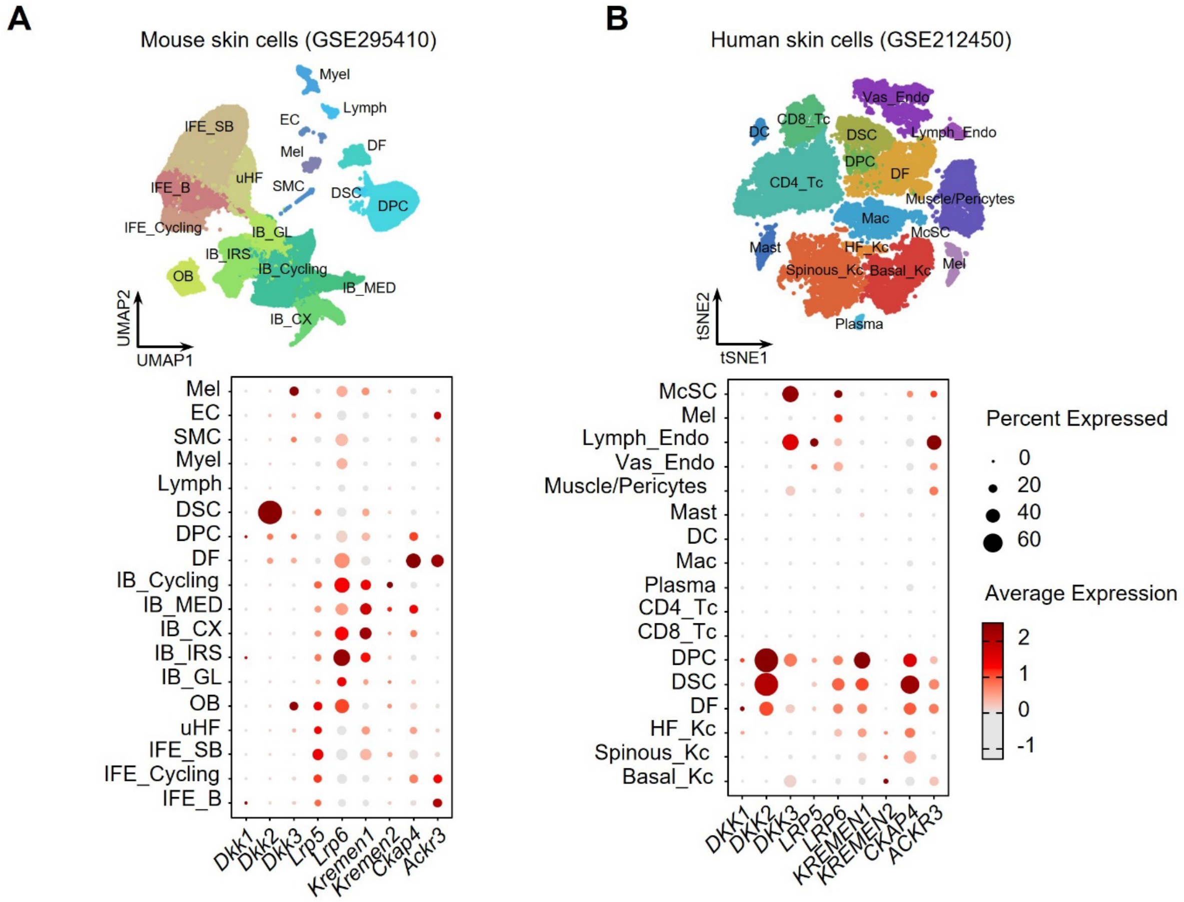

Figure S1. Expression of DKK families and their receptors in mouse (A) and human (B). Single-cell RNA-seq analysis of mouse skin (GSE295410) and human skin (GSE212450). Top: UMAP (mouse) and t-SNE (human) visualization showing annotated cell clusters. Bottom: Dot plot displaying the expression levels and proportion of cells expressing Dkk family genes (Dkk1-3) and known DKK receptors (Lrp5, Lrp6, Kremen1, Kremen2, Ckap4, Acrk3) across various cell types.

Supplementary Figure 2

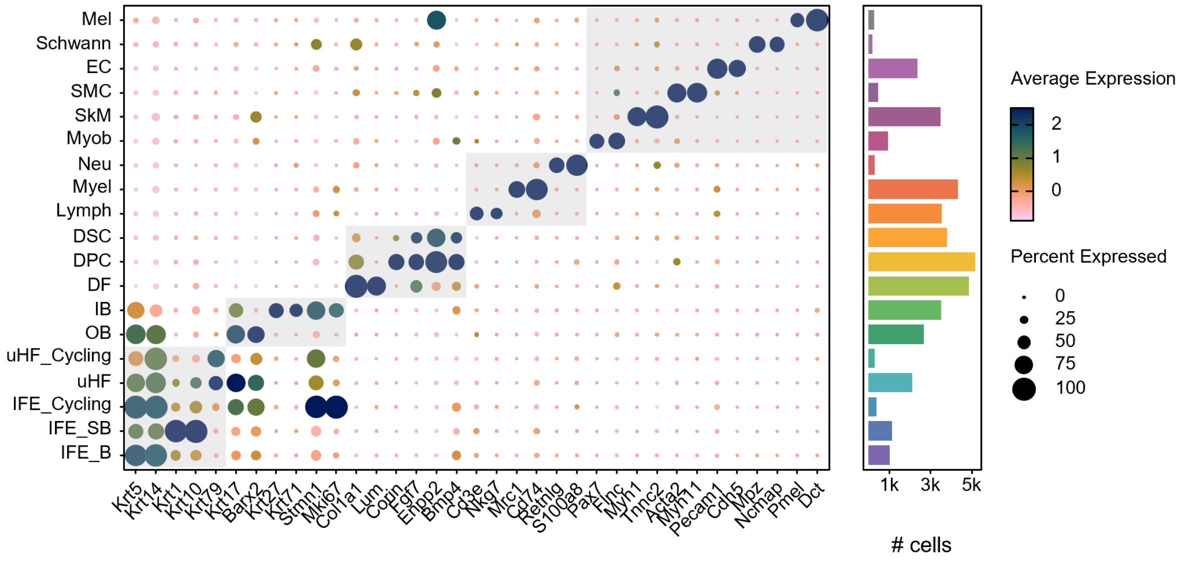

Figure S2. Marker gene expression defining the identified cell lineages. (Left) Dot plot displaying the expression of selected canonical marker genes used for cell-type annotation. The 19 major cell lineages correspond to the populations visualized in the UMAP of Figure 3A. The size of each dot represents the percentage of cells within a specific cluster expressing the indicated marker gene, and the color intensity reflects the average expression level (scaled). Gray shading highlights representative markers that uniquely define each population, including epithelial keratinocytes (IFE_B, IFE_SB, IFE_Cycling, uHF, uHF_Cycling, OB, IB), mesenchymal populations (DF, DPC, DSC), immune cells (Lymph, Myel, Neu), and other structural or niche-associated cell types (Myob, SkM, SMC, EC, Schwann, Mel). (Right) The bar graph indicates the total count of single cells recovered for each identified cell type, totaling 40,992 cells analyzed in the study.

Supplementary Figure 3

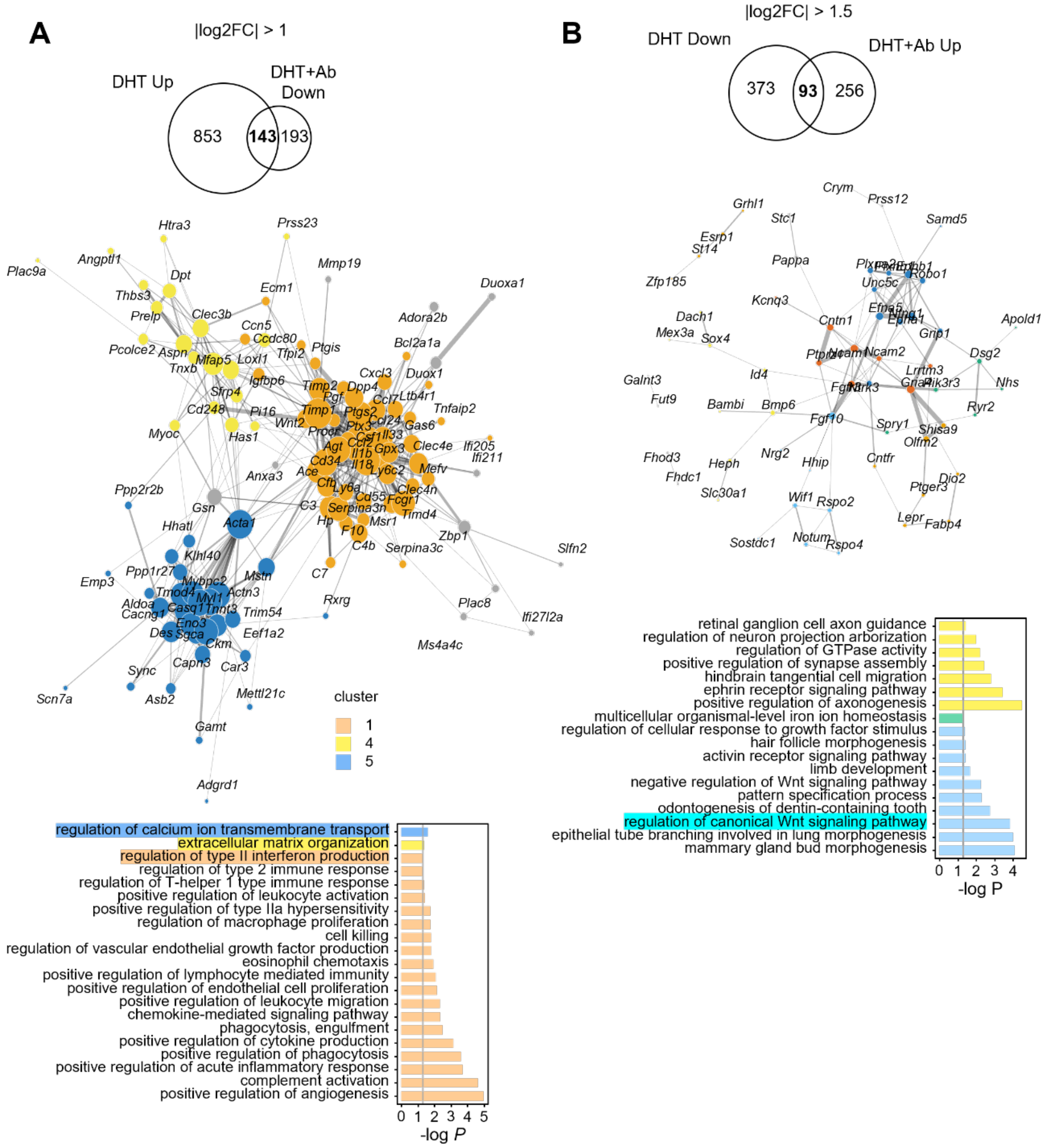

Figure S3. Functional enrichment of DEGs reversed by anti-DKK3 antibody treatment. **(A)** Identification and functional characterization of genes upregulated by DHT and downregulated by anti-DKK3 antibody. (Top) Venn diagram showing the overlap between genes significantly upregulated in DHT-treated skin (vs. Con) and those downregulated in DHT+Ab-treated skin (vs. DHT). (Middle) STRING network constructed from the shared DEGs. (Bottom) Gene Ontology (GO) biological process enrichment analysis performed for

each identified community. Representative terms with an adjusted $P < 0.05$ are shown. **(B)** Identification and functional characterization of genes downregulated by DHT and restored by anti-DKK3 antibody.

Supplementary Figure 4

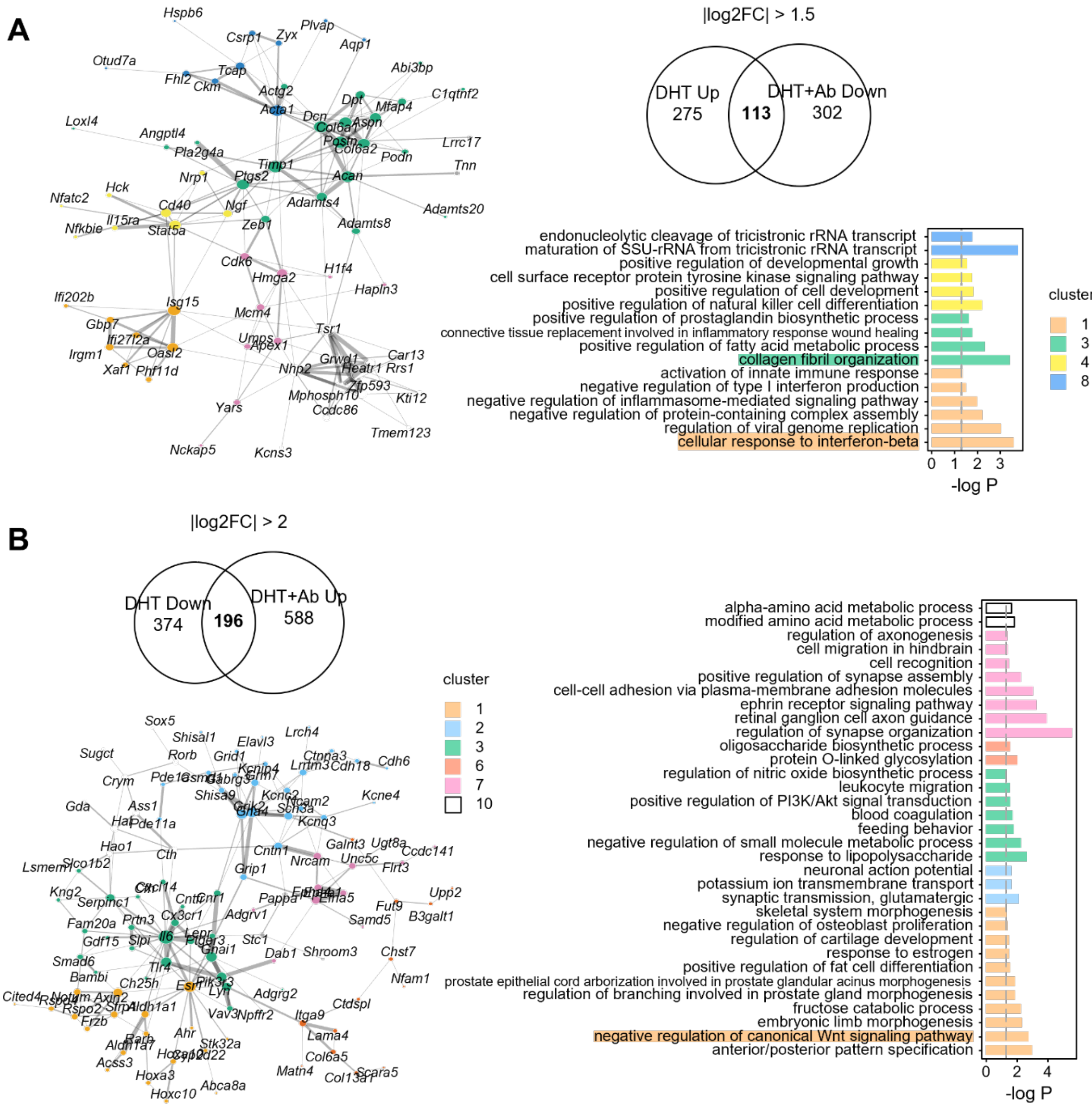

Figure S4. Functional enrichment of DEGs reversed by anti-DKK3 antibody treatment in DPCs. (A) Identification and functional characterization of genes upregulated by DHT and downregulated by anti-DKK3 antibody. **(B)** Identification and functional characterization of genes downregulated by DHT and restored by anti-DKK3 antibody.

Supplementary Figure 5

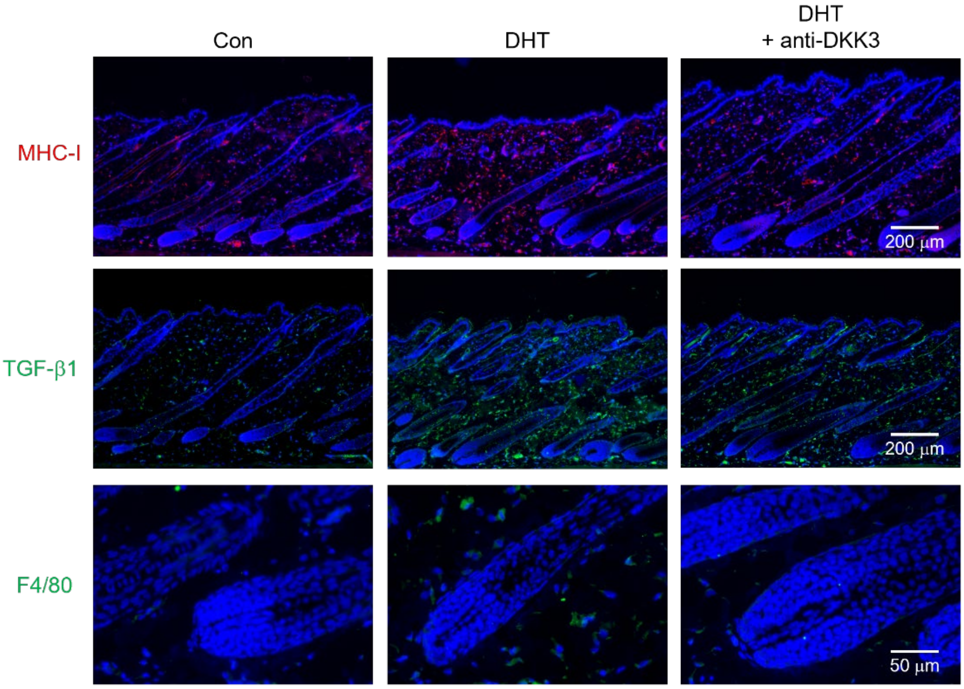

Figure S5. In vivo validation of fibroimmune remodeling in androgen-induced AGA mouse skin. Representative immunofluorescence images of dorsal skin sections from control, DHT-treated, and DHT plus anti-DKK3 antibody–treated mice. MHC-I (red) staining demonstrates increased immune activation in the dermal compartment following DHT treatment, which is reduced upon DKK3 neutralization. TGF-β1 (green) staining reveals enhanced extracellular matrix remodeling and fibroblast activation under androgen exposure, with decreased expression observed after anti-DKK3 antibody treatment. F4/80 (green) staining indicates increased macrophage infiltration in DHT-treated skin, which is attenuated by DKK3 blockade. Nuclei are counterstained with DAPI (blue). Scale bars: 200 μm (top and middle panels), 50 μm (bottom panels).

Supplementary Figure 6

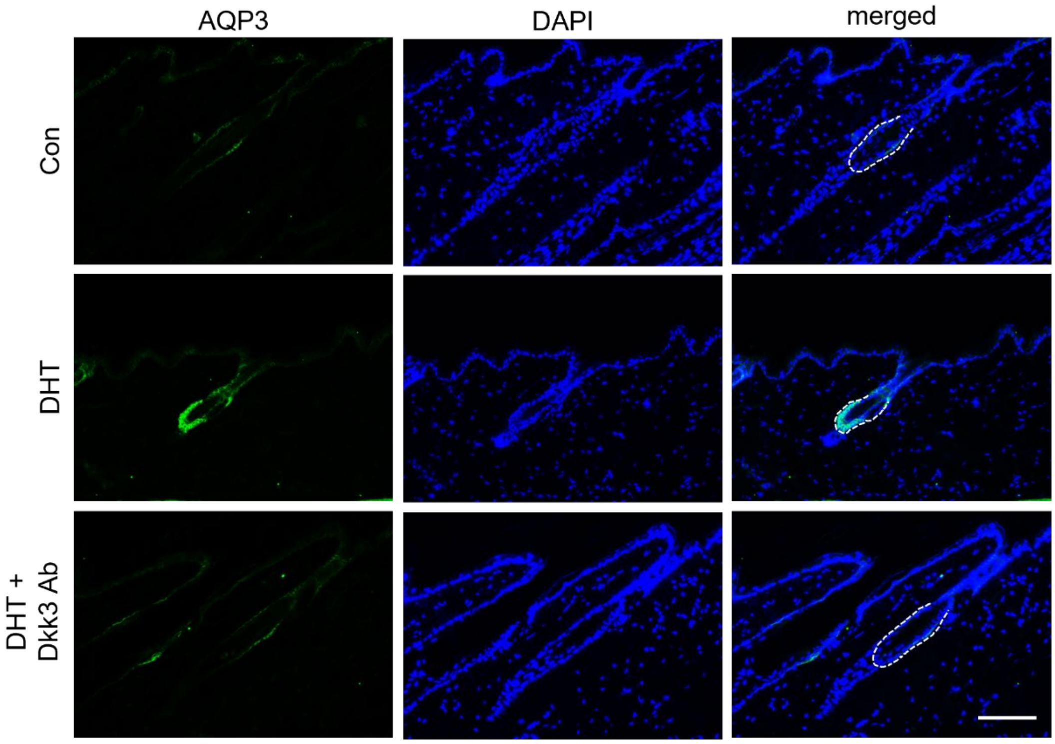

Figure S6. Increased expression of aquaporine3 (AQP3) in hair follicle keratinocytes of AGA model (DHT treatment), and its reversal after DKK3 antibody. Representative immunofluorescence images showing aquaporin 3 (AQP3, green) expression in mouse dorsal skin under control (Con), DHT-treated (DHT), and DHT plus anti-DKK3 antibody (DHT + DKK3 Ab) conditions. Nuclei are counterstained with DAPI (blue). Merged images highlight AQP3 expression in the hair follicle bulge region (white dashed outline). Scale bars, 100 μm.

Supplementary Figure 7

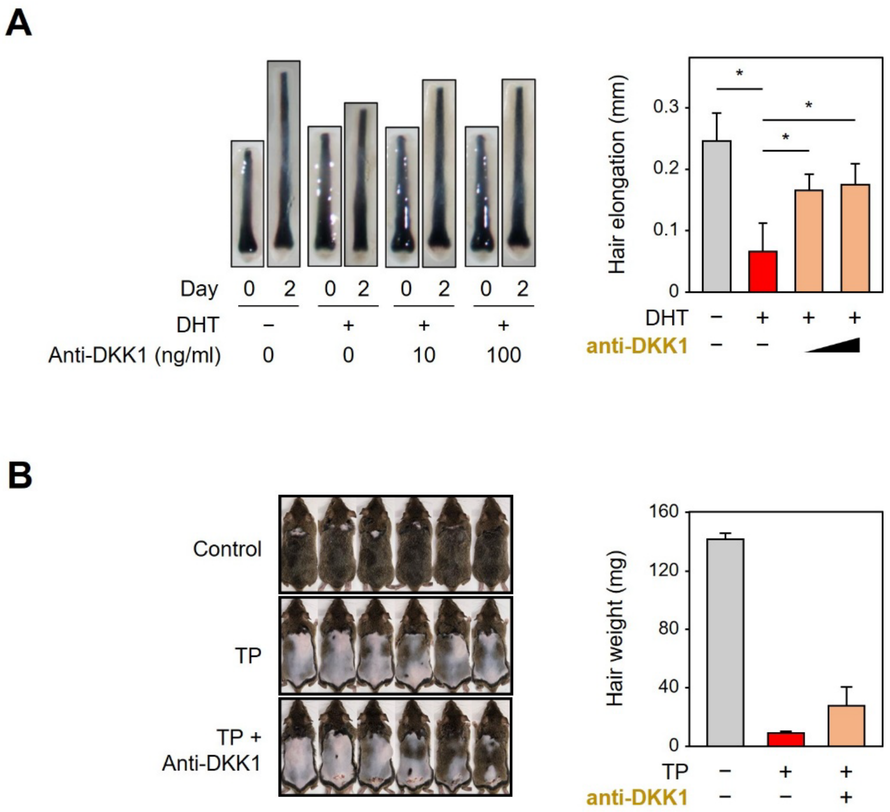

Figure S7. Hair growth promoting effect of DKK1 antibody. (A) In human hair follicle organ cultures, DHT (100 nM) suppressed hair shaft elongation, which was rescued by anti-DKK1 antibody treatment (10 and 100 ng/ml). $^{*}P < 0.05$. **(B)** Representative dorsal photographs and quantification of hair weight (mean ± s.e.m.) in mice treated with testosterone propionate (TP) with or without anti-DKK1 antibody.
